# Supplementary material for: Suboptimal infant and young child feeding practices in rural Boucle du Mouhoun, Burkina Faso: Findings from a cross-sectional population-based survey
Source: PLoS One. 2019 Nov 12;14(11):e0224769. doi: 10.1371/journal.pone.0224769 (PMC6850548; doi:10.1371/journal.pone.0224769)
Supplement: S1 Table — (DOCX) [file pone.0224769.s001.docx]

**S1 Table: Socio-demographic characteristics of interviewed mothers and care seeking at a health facility along the continuum of care (N = 2,229)**

|  |  | % | 95%CI | | |
| --- | --- | --- | --- | --- | --- |
| Mother's age | 15-24 years | **37.5** | 34.8 | 40.4 | |
|  | 25-34 years | **45.0** | 42.7 | 47.3 | |
|  | 35-49 years | **17.5** | 15.4 | 19.8 | |
| Mother's religion | Catholic/Protestant | **28.5** | 22.1 | 35.9 | |
|  | Muslim | **63.4** | 54.7 | 71.3 | |
|  | Animist/Atheist | **8.1** | 4.7 | 13.4 | |
| Mother's education level | None | **73.2** | 69.2 | 76.9 | |
|  | Primary only | **18.0** | 15.5 | 20.9 | |
|  | Secondary or higher | **8.7** | 7.0 | 10.7 | |
| Mother's income generating activities (cash or kind) | | **57.9** | 50.9 | 64.6 | |
| Mother's marital status | Monogamous union | **64.6** | 61.1 | 68.0 | |
|  | Polygamous union | **33.5** | 30.0 | 37.0 | |
|  | Single, divorced/separated, widow | **1.9** | 1.3 | 2.8 | |
| Partner's education level | None | **66.5** | 62.2 | 70.5 | |
|  | Primary only | **23.7** | 20.7 | 26.9 | |
|  | Secondary or higher | **7.9** | 6.3 | 9.9 | |
|  | Not in union | **1.9** | 1.3 | 2.8 | |
| In union with a partner earning an income in cash or kind | | **81.7** | 78.9 | 84.2 | |
| Parity (live births) (mean) |  | **3.9** | 3.7 | 4.0 | |
| At least one antenatal (ANC) visit | | **99.3** | 98.2 | 99.7 | |
| 4 or more ANC visits | | **59.6** | 55.0 | 64.0 | |
| Facility delivery | | **89.9** | 85.7 | 93.0 | |
| Postnatal care visit within one week of delivery (mother or baby) | | **41.6** | 34.2 | 49.4 | |
| Child's birth order | First live birth | **18.0** | 16.3 | 19.9 | |
|  | 2nd or 3rd live birth | **31.9** | 29.7 | 34.2 | |
|  | 4th to 6th live birth | **35.9** | 34.0 | 37.8 | |
|  | 7th or above live birth | **14.2** | 12.1 | 16.7 | |
| Child's gender | Boy | **52.5** | 50.0 | 54.9 | |
|  | Girl | **47.5** | 45.1 | 50.0 | |
| Child's age | 6-8 months | **17.7** | 16.5 | 18.8 | |
|  | 9-11 months | **14.4** | 13.3 | 15.5 | |
|  | 12-15 months | **27.2** | 24.5 | 30.0 | |
|  | 16-19 months | **23.1** | 20.9 | 25.5 | |
|  | 20-23 months | **17.6** | 15.5 | 20.0 | |
| At least one well-baby consultation (W-BC) attendance since birth | | **70.8** | 63.8 | 76.9 | |
| Timely W-BC† | | **21.8** | 17.6 | 26.6 | |
| At least one visit to a health facility for immunisation since birth | | **96.7** | 95.1 | 97.8 | |
| Fever, cough, fast/difficult breathing or diarrhoea (past 2 weeks) | | **37.0** | 32.4 | 42.0 | |
| Consultation for childhood illness (sick children, past 2 weeks) | | **60.9** | 54.9 | 66.5 | |
| Consultation for diarrhoea (sick children, past 2 weeks) | | **58.9** | 51.3 | 66.2 | |
| Household clean water source* | | **47.6** | 38.8 | 56.6 | |
| Time from water source | In the yard | **10.7** | 7.8 | 14.6 | |
|  | < 10 minutes | **28.5** | 25.0 | 32.3 | |
|  | 10 to 30 minutes | **41.7** | 38.2 | 45.2 | |
|  | > 30 minutes | **19.1** | 16.0 | 22.7 | |
| * Public fountain, borehole, tap water; † Last WBC within 1 month if 6-11 months old or 2 months if 12-23 months old | | | | |  |
