# Supplementary material for: Suboptimal infant and young child feeding practices in rural Boucle du Mouhoun, Burkina Faso: Findings from a cross-sectional population-based survey
Source: PLoS One. 2019 Nov 12;14(11):e0224769. doi: 10.1371/journal.pone.0224769 (PMC6850548; doi:10.1371/journal.pone.0224769)
Supplement: S2 Table — (DOCX) [file pone.0224769.s002.docx]

**S2 Table: Predictors of introduction of soft, semi-solid, solid foods (SSS) in children 6 to 23 months of age (N = 2,229)**

|  |  | N | SSS % | Univariable | | | | | Multivariable | | | | |
| --- | --- | --- | --- | --- | --- | --- | --- | --- | --- | --- | --- | --- | --- |
|  |  |  |  | OR | | 95%CI | | P-value | OR | 95%CI | | | P-value |
| Mother's age | 15-24 years | 836 | 88.2 | **1.00** | | - | - | 0.393 |  |  |  | |  |
|  | 25-34 years | 1,004 | 86.5 | **0.88** | | 0.66 | 1.17 |  |  |  |  | |  |
|  | 35-49 years | 389 | 88.6 | **1.06** | | 0.77 | 1.45 |  |  |  |  | |  |
| Mother's ethnicity* |  |  |  |  | |  |  | 0.548 |  |  |  | |  |
| Mother's religion | Catholic/Protestant | 635 | 87.3 | **1.00** | | - | - | 0.256 |  |  |  | |  |
|  | Muslim | 1,414 | 87.1 | **1.09** | | 0.80 | 1.49 |  |  |  |  | |  |
|  | Animist/Atheist | 180 | 92.1 | **1.72** | | 0.89 | 3.33 |  |  |  |  | |  |
| Mother's education level | None | 1,633 | 87.2 | **1.00** | | - | - | 0.699 |  |  |  | |  |
|  | Primary only | 402 | 87.8 | **1.03** | | 0.72 | 1.46 |  |  |  |  | |  |
|  | Secondary or higher | 194 | 90.0 | **1.27** | | 0.73 | 2.22 |  |  |  |  | |  |
| Mother's income generating activities (cash or kind) | No | 939 | 84.8 | **1.00** | | - | - | 0.011 | **1.00** | - | - | | 0.010 |
|  | Yes | 1,290 | 89.5 | **1.47** | | 1.09 | 1.98 |  | **1.59** | 1.12 | 2.26 | |  |
| Mother's marital status | Monogamous union | 1,441 | 87.4 | **1.00** | | - | - | 0.911 |  |  |  | |  |
|  | Polygamous union | 746 | 87.8 | **1.07** | | 0.78 | 1.48 |  |  |  |  | |  |
|  | Single, separated, widow | 43 | 88.0 | **1.04** | | 0.43 | 2.54 |  |  |  |  | |  |
| Partner's education level | None | 1,482 | 86.9 | **1.00** | | - | - | 0.767 |  |  |  | |  |
|  | Primary only | 528 | 88.5 | **1.13** | | 0.84 | 1.52 |  |  |  |  | |  |
|  | Secondary or higher | 177 | 90.0 | **1.22** | | 0.77 | 1.92 |  |  |  |  | |  |
|  | Not in union | 43 | 88.0 | **1.07** | | 0.44 | 2.61 |  |  |  |  | |  |
| In union with a partner earning an income in cash or kind | No | 408 | 85.0 | **1.00** | | - | - | 0.044 | **1.00** | - | - | | 0.107 |
|  | Yes | 1,821 | 88.1 | **1.37** | | 1.01 | 1.86 |  | **1.41** | 0.93 | 2.13 | |  |
| 4 or more ANC visits | No | 901 | 85.7 | **1.00** | | - | - | 0.200 |  |  |  | |  |
|  | Yes | 1,328 | 88.8 | **1.20** | | 0.91 | 1.59 |  |  |  |  | |  |
| Facility delivery | No | 225 | 83.2 | **1.00** | | - | - | 0.177 |  |  |  | |  |
|  | Yes | 2,004 | 88.0 | **1.34** | | 0.88 | 2.06 |  |  |  |  | |  |
| Postnatal care visit within 1 week of delivery (mother or baby) | No | 1,302 | 87.1 | **1.00** | | - | - | 0.988 |  |  |  | |  |
|  | Yes | 927 | 88.1 | **1.00** | | 0.79 | 1.27 |  |  |  |  | |  |
| Child's birth order | First live birth | 401 | 90.0 | **1.00** | | - | - | 0.218 |  |  |  | |  |
|  | 2nd or 3rd live birth | 711 | 86.5 | **0.70** | | 0.48 | 1.00 |  |  |  |  | |  |
|  | 4th to 6th live birth | 800 | 87.1 | **0.76** | | 0.50 | 1.13 |  |  |  |  | |  |
|  | 7th or above live birth | 317 | 87.8 | **0.83** | | 0.53 | 1.30 |  |  |  |  | |  |
| Child's gender | Boy | 1,170 | 87.4 | **1.00** | | - | - | 0.985 |  |  |  | |  |
|  | Girl | 1,059 | 87.6 | **1.00** | | 0.75 | 1.34 |  |  |  |  | |  |
| Child's age | 6-8 months | 394 | 54.1 | **1.00** | | - | - | < 0.001 | **1.00** | - | - | | < 0.001 |
|  | 9-11 months | 321 | 86.6 | **6.66** | | 4.69 | 9.46 |  | **6.99** | 5.04 | 9.68 | |  |
|  | 12-15 months | 605 | 92.8 | **14.31** | | 9.63 | 21.27 |  | **15.06** | 10.02 | 22.63 | |  |
|  | 16-19 months | 516 | 98.4 | **73.20** | | 34.39 | 155.82 |  | **76.19** | 36.25 | 160.10 | |  |
|  | 20-23 months | 393 | 99.3 | **153.80** | | 40.97 | 577.30 |  | **160.37** | 41.83 | 614.87 | |  |
| Fever, cough, fast/difficult breathing or diarrhoea (past 2 weeks) | No | 1,403 | 87.8 | **1.00** | | - | - | 0.658 |  |  |  | |  |
|  | Yes | 826 | 87.0 | **1.07** | | 0.79 | 1.45 |  |  |  |  | |  |
| At least one well-baby consultation (W-BC) attendance since birth | No | 651 | 83.8 | **1.00** | | - | - | 0.001 | **1.00** | - | - | | 0.679 |
|  | Yes | 1,578 | 89.1 | **1.51** | | 1.18 | 1.93 |  | **1.07** | 0.77 | 1.51 | |  |
| At least one visit to a health facility for immunisation since birth | No | 73 | 77.9 | **1.00** | | - | - | 0.005 | **1.00** | - | - | | 0.916 |
|  | Yes | 2,156 | 87.9 | **1.76** | | 1.19 | 2.61 |  | **0.96** | 0.45 | 2.04 | |  |
| Received facility-based information on complementary feeding | No | 1,039 | 85.1 | **1.00** | | - | - | 0.008 | **1.00** | - | - | | 0.256 |
|  | Yes | 1,189 | 89.7 | **1.42** | | 1.10 | 1.84 |  | **1.26** | 0.84 | 1.89 | |  |
| Received community-based information on complementary feeding | No | 1,510 | 86.0 | **1.00** | | - | - | < 0.001 | **1.00** | - | - | | 0.029 |
|  | Yes | 719 | 90.7 | **1.69** | | 1.35 | 2.13 |  | **1.49** | 1.04 | 2.12 | |  |
| Knowledge score of timely introduction of foods† | | 2,229 | - | **1.11** | | 1.02 | 1.22 | 0.021 | **1.08** | 0.95 | 1.23 | | 0.232 |
| Household wealth quintile | Poorest | 455 | 86.0 | **1.00** | | - | - | 0.795 |  |  |  | |  |
|  | Poorer | 436 | 86.9 | **1.08** | | 0.74 | 1.58 |  |  |  |  | |  |
|  | Middle | 435 | 86.6 | **1.01** | | 0.69 | 1.48 |  |  |  |  | |  |
|  | Richer | 446 | 88.8 | **1.19** | | 0.84 | 1.70 |  |  |  |  | |  |
|  | Richest | 449 | 89.1 | **1.16** | | 0.75 | 1.81 |  |  |  |  | |  |
| Household clean water source** | No | 1,168 | 85.7 | **1.00** | | - | - | 0.007 | **1.00** | - | - | | 0.179 |
|  | Yes | 1,061 | 89.5 | **1.38** | | 1.09 | 1.73 |  | **1.30** | 0.89 | 1.92 | |  |
| Time from water source | > 30 minutes | 427 | 85.4 | **1.00** | | - | - | 0.166 | **1.00** | - | - | | 0.190 |
|  | 10 to 30 minutes | 928 | 88.7 | **1.38** | | 0.97 | 1.96 |  | **1.49** | 0.97 | 2.28 | |  |
|  | < 10 minutes | 874 | 87.3 | **1.17** | | 0.84 | 1.65 |  | **1.39** | 0.90 | 2.15 | |  |
| * Only P-value shown to comply with the ethical requirement in Burkina Faso | | | | |  |  |  |  |  |  |  | |  |
| ** Public fountain, borehole, tap water; † At 6 months for water and other liquids, soft, semi-solid and solid foods, at 9 months for eggs and meat | | | | | | | | | | | |  |  |
