# Supplementary material for: Suboptimal infant and young child feeding practices in rural Boucle du Mouhoun, Burkina Faso: Findings from a cross-sectional population-based survey
Source: PLoS One. 2019 Nov 12;14(11):e0224769. doi: 10.1371/journal.pone.0224769 (PMC6850548; doi:10.1371/journal.pone.0224769)
Supplement: S3 Table — (DOCX) [file pone.0224769.s003.docx]

**S3 Table: Predictors of minimum dietary diversity (MDD) in children 6 to 23 months of age (N = 2,229)**

|  |  | N | MDD % | Univariable | | | | | Multivariable | | | |
| --- | --- | --- | --- | --- | --- | --- | --- | --- | --- | --- | --- | --- |
|  |  |  |  | OR | | 95%CI | | P-value | OR | 95%CI | | P-value |
| Mother's age | 15-24 years | 836 | 19.5 | **1.00** | | - | - | 0.530 |  |  |  |  |
|  | 25-34 years | 1,004 | 17.2 | **0.88** | | 0.68 | 1.13 |  |  |  |  |  |
|  | 35-49 years | 389 | 18.0 | **0.98** | | 0.66 | 1.43 |  |  |  |  |  |
| Mother's ethnicity* |  |  |  |  | |  |  | 0.023 |  |  |  | < 0.001 |
| Mother's religion | Catholic/Protestant | 635 | 15.8 | **1.00** | | - | - | 0.309 |  |  |  |  |
|  | Muslim | 1,414 | 19.0 | **1.43** | | 0.90 | 2.28 |  |  |  |  |  |
|  | Animist/Atheist | 180 | 20.7 | **1.23** | | 0.73 | 2.06 |  |  |  |  |  |
| Mother's education level | None | 1,633 | 18.1 | **1.00** | | - | - | 0.514 |  |  |  |  |
|  | Primary only | 402 | 19.7 | **1.10** | | 0.76 | 1.60 |  |  |  |  |  |
|  | Secondary or higher | 194 | 15.6 | **0.83** | | 0.47 | 1.45 |  |  |  |  |  |
| Mother's income generating activities (cash or kind) | No | 939 | 15.1 | **1.00** | | - | - | 0.013 | **1.00** | - | - | 0.224 |
|  | Yes | 1,290 | 20.4 | **1.39** | | 1.07 | 1.81 |  | **1.20** | 0.89 | 1.62 |  |
| Mother's marital status | Monogamous union | 1,441 | 18.9 | **1.00** | | - | - | 0.571 |  |  |  |  |
|  | Polygamous union | 746 | 17.2 | **0.92** | | 0.68 | 1.24 |  |  |  |  |  |
|  | Single, separated, widow | 43 | 12.8 | **0.64** | | 0.26 | 1.55 |  |  |  |  |  |
| Partner's education level | None | 1,482 | 18.5 | **1.00** | | - | - | 0.530 |  |  |  |  |
|  | Primary only | 528 | 17.2 | **0.94** | | 0.67 | 1.34 |  |  |  |  |  |
|  | Secondary or higher | 177 | 19.9 | **1.20** | | 0.70 | 2.05 |  |  |  |  |  |
|  | Not in union | 43 | 12.8 | **0.67** | | 0.28 | 1.57 |  |  |  |  |  |
| In union with a partner earning an income in cash or kind | No | 408 | 11.5 | **1.00** | | - | - | < 0.001 | **1.00** | - | - | 0.018 |
|  | Yes | 1,821 | 19.7 | **1.88** | | 1.33 | 2.67 |  | **1.67** | 1.09 | 2.54 |  |
| 4 or more ANC visits | No | 901 | 17.0 | **1.00** | | - | - | 0.738 |  |  |  |  |
|  | Yes | 1,328 | 19.0 | **1.05** | | 0.78 | 1.42 |  |  |  |  |  |
| Facility delivery | No | 225 | 16.8 | **1.00** | | - | - | 0.911 |  |  |  |  |
|  | Yes | 2,004 | 18.3 | **1.04** | | 0.53 | 2.03 |  |  |  |  |  |
| Postnatal care visit within 1 week of delivery (mother or baby) | No | 1,302 | 16.9 | **1.00** | | - | - | 0.349 |  |  |  |  |
|  | Yes | 927 | 20.0 | **1.14** | | 0.87 | 1.48 |  |  |  |  |  |
| Child's birth order | First live birth | 401 | 19.8 | **1.00** | | - | - | 0.956 |  |  |  |  |
|  | 2nd or 3rd live birth | 711 | 17.6 | **0.92** | | 0.61 | 1.40 |  |  |  |  |  |
|  | 4th to 6th live birth | 800 | 18.1 | **0.92** | | 0.66 | 1.28 |  |  |  |  |  |
|  | 7th or above live birth | 317 | 17.7 | **0.92** | | 0.57 | 1.48 |  |  |  |  |  |
| Child's gender | Boy | 1,170 | 16.9 | **1.00** | | - | - | 0.249 |  |  |  |  |
|  | Girl | 1,059 | 19.6 | **1.20** | | 0.88 | 1.62 |  |  |  |  |  |
| Child's age | 6-8 months | 394 | 3.6 | **1.00** | | - | - | < 0.001 | **1.00** | - | - | < 0.001 |
|  | 9-11 months | 321 | 10.4 | **3.45** | | 1.89 | 6.29 |  | **3.27** | 1.75 | 6.11 |  |
|  | 12-15 months | 605 | 17.8 | **6.72** | | 4.18 | 10.80 |  | **6.38** | 3.90 | 10.44 |  |
|  | 16-19 months | 516 | 25.6 | **10.63** | | 6.94 | 16.27 |  | **11.55** | 7.22 | 18.46 |  |
|  | 20-23 months | 393 | 30.1 | **15.76** | | 9.07 | 27.37 |  | **15.44** | 8.70 | 27.39 |  |
| Fever, cough, fast/difficult breathing or diarrhoea (past 2 weeks) | No | 1,403 | 18.2 | **1.00** | | - | - | 0.667 |  |  |  |  |
|  | Yes | 826 | 18.2 | **1.06** | | 0.82 | 1.36 |  |  |  |  |  |
| At least one well-baby consultation (W-BC) attendance since birth | No | 651 | 12.7 | **1.00** | | - | - | < 0.001 | **1.00** | - | - | 0.013 |
|  | Yes | 1,578 | 20.5 | **2.08** | | 1.41 | 3.07 |  | **1.75** | 1.13 | 2.73 |  |
| At least one visit to a health facility for immunisation since birth | No | 73 | 2.7 | **1.00** | | - | - | 0.018 | **1.00** | - | - | 0.091 |
|  | Yes | 2,156 | 18.7 | **6.45** | | 1.38 | 30.20 |  | **4.07** | 0.80 | 20.81 |  |
| Received facility-based information on complementary feeding | No | 1,039 | 15.5 | **1.00** | | - | - | 0.159 |  |  |  |  |
|  | Yes | 1,189 | 20.6 | **1.23** | | 0.92 | 1.64 |  |  |  |  |  |
| Received community-based information on complementary feeding | No | 1,510 | 15.0 | **1.00** | | - | - | 0.001 | **1.00** | - | - | 0.013 |
|  | Yes | 719 | 24.8 | **1.74** | | 1.27 | 2.37 |  | **1.55** | 1.10 | 2.20 |  |
| Household wealth quintile | Poorest | 455 | 11.7 | **1.00** | | - | - | 0.002 | **1.00** | - | - | 0.003 |
|  | Poorer | 436 | 14.4 | **1.13** | | 0.72 | 1.78 |  | **1.08** | 0.68 | 1.72 |  |
|  | Middle | 435 | 17.7 | **1.26** | | 0.69 | 2.29 |  | **1.12** | 0.62 | 2.05 |  |
|  | Richer | 446 | 22.4 | **1.81** | | 1.14 | 2.89 |  | **1.67** | 0.99 | 2.80 |  |
|  | Richest | 449 | 24.6 | **2.09** | | 1.24 | 3.53 |  | **2.06** | 1.21 | 3.50 |  |
| Household clean water source** | No | 1,168 | 14.8 | **1.00** | | - | - | 0.033 | **1.00** | - | - | 0.514 |
|  | Yes | 1,061 | 21.9 | **1.38** | | 1.03 | 1.85 |  | **1.12** | 0.79 | 1.59 |  |
| Time from water source | > 30 minutes | 427 | 17.1 | **1.00** | | - | - | 0.283 |  |  |  |  |
|  | 10 to 30 minutes | 928 | 16.5 | **1.05** | | 0.72 | 1.52 |  |  |  |  |  |
|  | < 10 minutes | 874 | 20.5 | **1.34** | | 0.89 | 2.03 |  |  |  |  |  |
| * Only P-value shown to comply with the ethical requirement in Burkina Faso | | | | |  |  |  |  |  |  |  |  |
| ** Public fountain, borehole, tap water | |  |  |  | |  |  |  |  |  |  |  |
