# Supplementary material for: Suboptimal infant and young child feeding practices in rural Boucle du Mouhoun, Burkina Faso: Findings from a cross-sectional population-based survey
Source: PLoS One. 2019 Nov 12;14(11):e0224769. doi: 10.1371/journal.pone.0224769 (PMC6850548; doi:10.1371/journal.pone.0224769)
Supplement: S4 Table — (DOCX) [file pone.0224769.s004.docx]

**S4 Table: Predictors of minimum meal frequency (MMF) in children 6 to 23 months of age (N = 1,971)**

|  |  | N | MMF % | Univariable | | | | | Multivariable | | | |
| --- | --- | --- | --- | --- | --- | --- | --- | --- | --- | --- | --- | --- |
|  |  |  |  | OR | | 95%CI | | P-value | OR | 95%CI | | P-value |
| Mother's age | 15-24 years | 760 | 61.1 | **1.00** | | - | - | 0.715 |  |  |  |  |
|  | 25-34 years | 876 | 58.4 | **0.91** | | 0.70 | 1.17 |  |  |  |  |  |
|  | 35-49 years | 334 | 60.8 | **0.90** | | 0.65 | 1.24 |  |  |  |  |  |
| Mother's ethnicity* |  |  |  |  | |  |  | 0.508 |  |  |  |  |
| Mother's religion | Catholic/Protestant | 574 | 58.5 | **1.00** | | - | - | 0.761 |  |  |  |  |
|  | Muslim | 1,254 | 61.1 | **1.00** | | 0.75 | 1.33 |  |  |  |  |  |
|  | Animist/Atheist | 143 | 53.9 | **0.87** | | 0.56 | 1.35 |  |  |  |  |  |
| Mother's education level | None | 1,422 | 58.8 | **1.00** | | - | - | 0.281 |  |  |  |  |
|  | Primary only | 364 | 60.0 | **1.06** | | 0.79 | 1.42 |  |  |  |  |  |
|  | Secondary or higher | 185 | 67.4 | **1.44** | | 0.92 | 2.27 |  |  |  |  |  |
| Mother's income generating activities (cash or kind) | No | 812 | 54.3 | **1.00** | | - | - | < 0.001 | **1.00** | - | - | 0.006 |
|  | Yes | 1,159 | 63.7 | **1.56** | | 1.22 | 2.00 |  | **1.42** | 1.11 | 1.82 |  |
| Mother's marital status | Monogamous union | 1,302 | 60.4 | **1.00** | | - | - | 0.576 |  |  |  |  |
|  | Polygamous union | 630 | 58.3 | **0.89** | | 0.72 | 1.11 |  |  |  |  |  |
|  | Single, separated, widow | 39 | 66.3 | **1.04** | | 0.43 | 2.50 |  |  |  |  |  |
| Partner's education level | None | 1,299 | 58.4 | **1.00** | | - | - | 0.761 |  |  |  |  |
|  | Primary only | 474 | 62.6 | **1.15** | | 0.88 | 1.52 |  |  |  |  |  |
|  | Secondary or higher | 159 | 61.5 | **1.07** | | 0.67 | 1.70 |  |  |  |  |  |
|  | Not in union | 39 | 66.3 | **1.13** | | 0.47 | 2.71 |  |  |  |  |  |
| In union with a partner earning an income in cash or kind | No | 370 | 55.0 | **1.00** | | - | - | 0.020 | **1.00** | - | - | 0.088 |
|  | Yes | 1,601 | 60.9 | **1.57** | | 1.07 | 2.30 |  | **1.46** | 0.94 | 2.26 |  |
| 4 or more ANC visits | No | 795 | 59.0 | **1.00** | | - | - | 0.733 |  |  |  |  |
|  | Yes | 1,176 | 60.4 | **0.96** | | 0.76 | 1.21 |  |  |  |  |  |
| Facility delivery | No | 195 | 52.1 | **1.00** | | - | - | 0.906 |  |  |  |  |
|  | Yes | 1,776 | 60.7 | **1.02** | | 0.72 | 1.44 |  |  |  |  |  |
| Postnatal care visit within 1 week of delivery (mother or baby) | No | 1,175 | 59.5 | **1.00** | | - | - | 0.811 |  |  |  |  |
|  | Yes | 796 | 60.3 | **1.03** | | 0.79 | 1.35 |  |  |  |  |  |
| Child's birth order | First live birth | 362 | 62.9 | **1.00** | | - | - | 0.684 |  |  |  |  |
|  | 2nd or 3rd live birth | 640 | 58.7 | **0.84** | | 0.61 | 1.16 |  |  |  |  |  |
|  | 4th to 6th live birth | 703 | 60.3 | **0.94** | | 0.66 | 1.33 |  |  |  |  |  |
|  | 7th or above live birth | 265 | 57.0 | **0.83** | | 0.56 | 1.22 |  |  |  |  |  |
| Child's gender | Boy | 1,045 | 57.6 | **1.00** | | - | - | 0.070 | **1.00** | - | - | 0.027 |
|  | Girl | 926 | 62.4 | **1.27** | | 0.98 | 1.65 |  | **1.34** | 1.03 | 1.73 |  |
| Child's age | 6-8 months | 369 | 40.3 | **1.00** | | - | - | < 0.001 | **1.00** | - | - | < 0.001 |
|  | 9-11 months | 284 | 49.7 | **1.50** | | 1.03 | 2.17 |  | **1.45** | 0.99 | 2.11 |  |
|  | 12-15 months | 531 | 58.2 | **2.13** | | 1.56 | 2.91 |  | **2.09** | 1.54 | 2.84 |  |
|  | 16-19 months | 446 | 75.3 | **5.30** | | 3.41 | 8.25 |  | **5.44** | 3.48 | 8.52 |  |
|  | 20-23 months | 342 | 71.7 | **4.29** | | 2.90 | 6.35 |  | **4.47** | 3.02 | 6.62 |  |
| Fever, cough, fast/difficult breathing or diarrhoea (past 2 weeks) | No | 1,253 | 62.1 | **1.00** | | - | - | 0.664 |  |  |  |  |
|  | Yes | 717 | 55.8 | **0.95** | | 0.75 | 1.20 |  |  |  |  |  |
| At least one well-baby consultation (W-BC) attendance since birth | No | 579 | 53.1 | **1.00** | | - | - | 0.001 | **1.00** | - | - | 0.161 |
|  | Yes | 1,392 | 62.6 | **1.54** | | 1.21 | 1.96 |  | **1.18** | 0.94 | 1.49 |  |
| At least one visit to a health facility for immunisation since birth | No | 68 | 44.9 | **1.00** | | - | - | 0.397 |  |  |  |  |
|  | Yes | 1,903 | 60.4 | **1.28** | | 0.72 | 2.26 |  |  |  |  |  |
| Received facility-based information on complementary feeding | No | 910 | 52.3 | **1.00** | | - | - | < 0.001 | **1.00** | - | - | < 0.001 |
|  | Yes | 1,060 | 66.4 | **1.74** | | 1.36 | 2.21 |  | **1.64** | 1.25 | 2.15 |  |
| Received community-based information on complementary feeding | No | 1,324 | 56.9 | **1.00** | | - | - | 0.001 | **1.00** | - | - | 0.108 |
|  | Yes | 647 | 65.8 | **1.55** | | 1.20 | 2.01 |  | **1.29** | 0.95 | 1.75 |  |
| Correct knowledge of minimum meal frequency☨ | No | 682 | 50.7 | **1.00** | | - | - | < 0.001 | **1.00** | - | - | 0.001 |
|  | Yes | 1,289 | 64.7 | **1.57** | | 1.24 | 1.99 |  | **1.59** | 1.21 | 2.10 |  |
| Household wealth quintile | Poorest | 401 | 55.9 | **1.00** | | - | - | 0.314 |  |  |  |  |
|  | Poorer | 387 | 60.7 | **1.25** | | 0.93 | 1.69 |  |  |  |  |  |
|  | Middle | 375 | 61.9 | **1.36** | | 0.99 | 1.85 |  |  |  |  |  |
|  | Richer | 393 | 61.0 | **1.24** | | 0.90 | 1.71 |  |  |  |  |  |
|  | Richest | 409 | 59.3 | **1.19** | | 0.78 | 1.81 |  |  |  |  |  |
| Household clean water source** | No | 1,020 | 59.6 | **1.00** | | - | - | 0.323 |  |  |  |  |
|  | Yes | 951 | 60.1 | **1.12** | | 0.89 | 1.41 |  |  |  |  |  |
| Time from water source | > 30 minutes | 389 | 52.3 | **1.00** | | - | - | 0.049 | **1.00** | - | - | 0.042 |
|  | 10 to 30 minutes | 828 | 63.0 | **1.58** | | 0.97 | 1.95 |  | **1.65** | 1.11 | 2.45 |  |
|  | < 10 minutes | 754 | 60.3 | **1.37** | | 1.09 | 2.30 |  | **1.42** | 0.97 | 2.06 |  |
| * Only P-value shown to comply with the ethical requirement in Burkina Faso | | | | |  |  |  |  |  |  |  |  |
| ** Public fountain, borehole, tap water;☨2 times if 6-8 months, 3 times if 9-23 months | | | | | |  |  |  |  |  |  |  |
