# Supplementary material for: Suboptimal infant and young child feeding practices in rural Boucle du Mouhoun, Burkina Faso: Findings from a cross-sectional population-based survey
Source: PLoS One. 2019 Nov 12;14(11):e0224769. doi: 10.1371/journal.pone.0224769 (PMC6850548; doi:10.1371/journal.pone.0224769)
Supplement: S1 Fig — (DOCX) [file pone.0224769.s005.docx]

**S1 Fig: Information on child complementary feeding received at a health facility (left) and in the community (right) (N = 2,229)**
