# Supplementary material for: Suboptimal infant and young child feeding practices in rural Boucle du Mouhoun, Burkina Faso: Findings from a cross-sectional population-based survey
Source: PLoS One. 2019 Nov 12;14(11):e0224769. doi: 10.1371/journal.pone.0224769 (PMC6850548; doi:10.1371/journal.pone.0224769)
Supplement: Attachment — Submitted filename: PLOSONE-D-19-20243_reviewer SG.pdf [file pone.0224769.s006.pdf]

PLOS ONE

Suboptimal infant and young child feeding practices in rural Burkina Faso: Findings from a cross-sectional population-based survey

--Manuscript Draft--

Manuscript Number:	PONE-D-19-20243
Article Type:	Research Article
Full Title:	Suboptimal infant and young child feeding practices in rural Burkina Faso: Findings from a cross-sectional population-based survey
Short Title:	Suboptimal infant and young child feeding practices in rural Burkina Faso
Corresponding Author:	Sophie Sarrassat<br>London School of Hygiene and Tropical Medicine<br>London, UNITED KINGDOM
Keywords:	Infant and young child feeding practices<br>Burkina Faso
Abstract:	Background<br>In Burkina Faso in 2016, 27% and 8% of children under-5 were estimated to suffer from stunting and wasting respectively. Here, we report on infant and young child feeding (IYCF) practices in rural areas of the Boucle du Mouhoun region.<br><br>Methods<br>A cross-sectional population-based survey was performed in 2017 in a representative sample of mothers of children aged 6 to 23 months. Selected IYCF practices were assessed using 24-hour dietary recall. Logistic regression was used to identify predictors of IYCF practices. All analyses accounted for sampling stratification by child's age group and for data clustering.<br><br>Findings<br>2,229 mothers were interviewed. According to them, 60% (95%CI 55, 65%) had received the minimum meal frequency, but only 18% (95%CI 15, 22%) and 13% (95%CI 10, 16%) benefited from the minimum dietary diversity and the minimum acceptable diet respectively. Only 16% (95%CI 13, 20%) of mothers reported increasing breastfeeding or liquids and continued feeding during an episode of child illness. Knowledge of timely introduction of complementary foods and recommended feeding practices during an illness were low. Despite positive attitudes towards the introduction of key food groups, mother's perceived self-efficacy to provide children with these food groups every day was relatively low.<br><br>Discussion<br>Our findings highlight the need for interventions to improve mothers' knowledge and practices in relation to IYCF in the Boucle du Mouhoun region. Behaviour change communication strategies have the potential to improve IYCF indicators, but sustained practices and optimal child growth may also require more practical support such as nutrition-sensitive interventions.
Order of Authors:	Sophie Sarrassat
	Rasmane Ganaba
	Henri Some
	Jenny Cresswell
	Hama Diallo
	Simon Cousens

Commented [WU1]: Needs to be deleted.

Commented [WU2]: What is the justification for selection of this area please?

Deleted: a child

Commented [WU3]: This is not the only study tool.

Commented [WU4]: Should be in words.

Deleted: their reports

Commented [WU5]: ?

Commented [WU6]: Only recommendations are presented in this section. Recommendations should be based only on study findings.

Powered by Editorial Manager® and ProduXion Manager® from Aries Systems Corporation

	Veronique Filippi
Additional Information:	
Question	Response

Financial Disclosure<br><br>Enter a financial disclosure statement that describes the sources of funding for the work included in this submission. Review the submission guidelines for detailed requirements. View published research articles from PLOS ONE for specific examples.<br><br>This statement is required for submission and will appear in the published article if the submission is accepted. Please make sure it is accurate.<br><br>Unfunded studies<br>Enter: The author(s) received no specific funding for this work.<br><br>Funded studies<br>Enter a statement with the following details:<br>• Initials of the authors who received each award<br>• Grant numbers awarded to each author<br>• The full name of each funder<br>• URL of each funder website<br>• Did the sponsors or funders play any role in the study design, data collection and analysis, decision to publish, or preparation of the manuscript?<br>• NO - Include this sentence at the end of your statement: The funders had no role in study design, data collection and analysis, decision to publish, or preparation of the manuscript.<br>• YES - Specify the role(s) played.<br><br>* typeset	Alive & Thrive is funded by the Bill & Melinda Gates Foundation and the governments of Canada and Ireland and managed by FHI 360. This research was supported by funding from the Bill & Melinda Gates Foundation, Grant Number OPP50838.

Powered by Editorial Manager® and ProduXion Manager® from Aries Systems Corporation

Competing Interests<br><br>Use the instructions below to enter a competing interest statement for this submission. On behalf of all authors, disclose any competing interests that could be perceived to bias this work—acknowledging all financial support and any other relevant financial or nonfinancial competing interests.<br><br>This statement will appear in the published article if the submission is accepted. Please make sure it is accurate. View published research articles from PLOS ONE for specific examples.<br><br>NO authors have competing interests<br><br>Enter: The authors have declared that no competing interests exist.<br><br>Authors with competing interests<br><br>Enter competing interest details beginning with this statement:<br><br>I have read the journal's policy and the authors of this manuscript have the following competing interests: [insert competing interests here]<br><br>* typeset	The authors have declared that no competing interests exist.

Powered by Editorial Manager® and ProduXion Manager® from Aries Systems Corporation

Ethics Statement<br><br>Enter an ethics statement for this submission. This statement is required if the study involved:<br><br>• Human participants<br>• Human specimens or tissue<br>• Vertebrate animals or cephalopods<br>• Vertebrate embryos or tissues<br>• Field research<br><br>Write "N/A" if the submission does not require an ethics statement.<br><br>General guidance is provided below. Consult the submission guidelines for detailed instructions. Make sure that all information entered here is included in the Methods section of the manuscript.	Ethical approval was granted by the National Health Ethic Committee of the Ministry of<br>Health of Burkina Faso (Reference 2015-5-061), the institutional review board of Centre MURAZ (Reference 2015-017) and the London School of Hygiene and Tropical Medicine (Reference 9066). Written informed consent was obtained prior to interview.

Powered by Editorial Manager® and ProduXion Manager® from Aries Systems Corporation

Format for specific study types<br><br>Human Subject Research (involving human participants and/or tissue)<br>• Give the name of the institutional review board or ethics committee that approved the study<br>• Include the approval number and/or a statement indicating approval of this research<br>• Indicate the form of consent obtained (written/oral) or the reason that consent was not obtained (e.g. the data were analyzed anonymously)<br><br>Animal Research (involving vertebrate animals, embryos or tissues)<br>• Provide the name of the Institutional Animal Care and Use Committee (IACUC) or other relevant ethics board that reviewed the study protocol, and indicate whether they approved this research or granted a formal waiver of ethical approval<br>• Include an approval number if one was obtained<br>• If the study involved non-human primates, add additional details about animal welfare and steps taken to ameliorate suffering<br>• If anesthesia, euthanasia, or any kind of animal sacrifice is part of the study, include briefly which substances and/or methods were applied<br><br>Field Research<br><br>Include the following details if this study involves the collection of plant, animal, or other materials from a natural setting:<br>• Field permit number<br>• Name of the institution or relevant body that granted permission	
Data Availability<br><br>Authors are required to make all data underlying the findings described fully available, without restriction, and from the time of publication. PLOS allows rare exceptions to address legal and ethical concerns. See the PLOS Data Policy and FAQ for detailed information.	Yes - all data are fully available without restriction. This contradicts the statement below.

Formatted: Centered

Powered by Editorial Manager® and ProduXion Manager® from Aries Systems Corporation

A Data Availability Statement describing where the data can be found is required at submission. Your answers to this question constitute the Data Availability Statement and will be published in the article, if accepted.<br><br>Important: Stating ‘data available on request from the author’ is not sufficient. If your data are only available upon request, select ‘No’ for the first question and explain your exceptional situation in the text box.<br><br>Do the authors confirm that all data underlying the findings described in their manuscript are fully available without restriction?	

Powered by Editorial Manager® and ProduXion Manager® from Aries Systems Corporation

Describe where the data may be found in full sentences. If you are copying our sample text, replace any instances of XXX with the appropriate details.<br><br>• If the data are held or will be held in a public repository, include URLs, accession numbers or DOIs. If this information will only be available after acceptance, indicate this by ticking the box below. For example: All XXX files are available from the XXX database (accession number(s) XXX, XXX.).<br>• If the data are all contained within the manuscript and/or Supporting Information files, enter the following: All relevant data are within the manuscript and its Supporting Information files.<br>• If neither of these applies but you are able to provide details of access elsewhere, with or without limitations, please do so. For example:<br><br>Data cannot be shared publicly because of [XXX]. Data are available from the XXX Institutional Data Access / Ethics Committee (contact via XXX) for researchers who meet the criteria for access to confidential data.<br><br>The data underlying the results presented in the study are available from (include the name of the third party and contact information or URL).<br>• This text is appropriate if the data are owned by a third party and authors do not have permission to share the data.<br><br>* typeset	Due to ethical restrictions, the full dataset will be made available to bona fide researchers on condition that they provide evidence of ethics approval and sign a Data Transfer Agreement. Further information on the data and access conditions can be found through the LSHTM Data Compass at: https://doi.org/10.17037/DATA.280.
Additional data availability information:	

Manuscript Click here to download Manuscript po_iycf practices_v5.docx

Full title: Suboptimal infant and young child feeding practices in rural Burkina Faso: Findings from a cross-sectional population-based survey

Short title: Suboptimal infant and young child feeding practices in rural Burkina Faso

Sarrassat S*, Ganaba R, Some H, Cresswell JA, Diallo AH, Cousens S, Filippi V

Authors' names, degree, affiliation, and full address

Department of Infectious Disease Epidemiology, London School of Hygiene and Tropical Medicine, London, UK (Dr Sophie Sarrassat, PhD; Dr Jenny A Cresswell, PhD; Prof Simon Cousens, MA; Pr Veronique Filippi, PhD); **Africsanté, Bobo-Dioulasso, Burkina Faso** (Dr Rasmane Ganaba, PhD; Henri Somé, BSc); **Centre Muraz, Bobo-Dioulasso, Burkina Faso** (Dr Abdoulaye Hama Diallo, PhD)

* Correspondence to: Dr Sophie Sarrassat, Centre for Maternal Adolescent Reproductive and Child Health (MARCH), London School of Hygiene and Tropical Medicine, Keppel Street, WC1E 7HT, London, UK; sophie.sarrassat@lshtm.ac.uk; 00 44 (0)20 7958 8198

Word counts

Abstract: 250 words

Full text: 4,024 words

Number of tables:8

Numberr of figures: 2

Number of references: 23

Abstract

Background

In Burkina Faso in 2016, 27% and 8% of children under-5 were estimated to suffer from stunting and wasting respectively. Here, we report on infant and young child feeding (IYCF) in rural areas of the Boucle du Mouhoun region.

Methods

A cross-sectional population-based survey was performed in 2017 in a representative sample of mothers of a child aged 6 to 23 months. IYCF practices were assessed using 24-hour dietary recall. Logistic regression was used to identify predictors of IYCF practices. All analyses accounted for sampling stratification by child's age group and for data clustering.

Findings

2,229 mothers were interviewed. According to their reports, 60% (95%CI 55, 65%) received the minimum meal frequency, but only 18% (95%CI 15, 22%) and 13% (95%CI 10, 16%) benefited from the minimum dietary diversity and the minimum acceptable diet respectively. Only 16% (95%CI 13, 20%) of mothers reported increasing breastfeeding or liquids and continued feeding during an episode of child illness. Knowledge of timely introduction of complementary foods and recommended feeding practices during an illness were low. Despite positive attitudes towards the introduction of key food groups, mother's perceived self-efficacy to provide children with these food groups every day was relatively low.

Discussion

Our findings highlight the need for interventions to improve mothers' knowledge and practices in relation to IYCF in the Boucle du Mouhoun region. Behaviour change communication strategies have the potential to improve IYCF indicators, but sustained practices and optimal child growth may also require more practical support such as nutrition-sensitive interventions.

Key words

Infant and young child feeding practices, Burkina Faso

Background

In 2011, under-nutrition, consisting of foetal growth restriction, stunting, wasting, and deficiencies of vitamin A and zinc, along with suboptimal breastfeeding, was estimated to underlie about 3.1 million under-five deaths, corresponding to 45% of all deaths in this age group (1). Improvements in nutrition will therefore be essential to the achievement of the Sustainable Development Goal (SDG) target 3.2 of 25 or fewer under-five deaths per 1,000 livebirths by 2030. To this end, the SDG targets 2.1 and 2.2 call respectively "*to end hunger and ensure access by all people to safe, nutritious and sufficient food all year round*" and "*to end all forms of malnutrition, achieving, by 2025, the internationally agreed targets on stunting and wasting in children under five years of age*" (2).

In Burkina Faso, despite a 56% decline, from an estimated 202 deaths per 1,000 live births in 1990 to 89 deaths per 1,000 live births in 2015, the under-5 mortality rate did not reach the 2015 Millennium Development Goal (MDG) target of 67 per 1,000 live births (3). In 2016, 20% of the total population was undernourished and 27% and 8% of under-5 children were estimated to suffer from stunting and wasting respectively (2).

Briefly mention the importance of IYCF practices in tackling malnutrition.

From December 2015 to July 2017, the Alive and Thrive (A&T) initiative sought to improve breastfeeding practices in rural communes in the Boucle du Mouhoun region of Burkina Faso. The intervention included interpersonal communication activities and community mobilisation activities. The evaluation used a repeated cross-sectional cluster randomised controlled trial design, and after 14 months of full implementation, selfreported exclusive breastfeeding increased between baseline and endline surveys by 36% points more in the intervention arm compared to the control arm (4).

Baseline findings highlighted suboptimal infant and young child feeding (IYCF) practices in children 6 to 11 months old (5), but no data were collected in children aged 12 months or more. Here, we report on IYCF practices of mothers of children aged 6 to 23 months, a group not targeted by the A&T initiative, using data collected during the endline survey.

Methods

Setting

Boucle du Mouhoun is one of the 13 regions of Burkina Faso, located in the north-west of the country. In this region, most of the population live in rural areas, largely dependent on subsistence agriculture, and the prevalences of wasting, stunting and underweight among under-five children were estimated at 9%, 23% and 17% respectively in 2016 (6).

Boucle du Mouhoun is divided into six health districts, five with a district hospital and one with a regional hospital. In rural areas, primary health facilities, run by nurses, are the most common point of care. In all public health facilities, free antenatal care (ANC), and subsidies for childbirth and emergency obstetric and neonatal care (EmONC) are provided. At the first level of care, growth monitoring and nutrition counselling, vitamin A supplementation and deworming are provided during under-five consultations through the Integrated Management of Childhood Illness (IMCI) strategy, preventive well-baby consultations (W-BC) and outreach activities. Briefly mention the utilization of these services.

Data collection

We performed a cross-sectional population-based survey in June and July 2017 in the 37 rural communes included in the trial, 18 of which had received the A+T intervention aimed at breastfeeding practices in children less than 6 months of age (5). Women of reproductive age, resident in the study area and mothers of a child aged 6 to 23 months living with them, were randomly selected using a two-stage sampling procedure. In each commune, three villages were first drawn with probability proportional to size using the most recent census (2006) as a sampling frame. At the second stage, eligible women living in selected villages were enumerated, and 20 mother-infant pairs (of whom 10 infants were 6 to 11 months old and 10 infants were 12 to 23 months old) were sampled per village using simple random sampling stratified on child age group.

Interviews were performed in local languages using a structured questionnaire programmed into electronic devices. Mothers were interviewed on their knowledge, attitudes, perceived self-efficacy and practices related to IYCF, and their care seeking behaviours related to maternal and child health. For each relevant contact reported in the community or in a health facility, women were also asked whether they received information on

Commented [WU7]: Broader than the scope of this study.

breastfeeding or child complementary feeding. IYCF practices were assessed using 24-hour dietary recall including a list of 29 liquids, soft, semi-solid and solid foods. Mothers were also questioned on the number of times they fed their child with soft, semi-solid or solid foods all together. Knowledge questions addressed timely introduction of seven key food groups, meal frequency and feeding practices during childhood illnesses. Attitudes towards IYCF were assessed by asking women whether they were in agreement with a set of statements. Perceived self-efficacy was assessed by asking women whether they felt capable of giving daily key food groups to their child.

The data collection involved 56 fieldworkers who were deployed in teams of six interviewers and one supervisor. Re-interviews were requested in case of incompleteness and/or inconsistencies, and all re-interviews were completed. Prior to the survey, fieldworkers received two-weeks training, including role-play in the four main languages spoken during interviews, and pilot surveys were performed in villages located outside study areas.

Sample size

Commented [WU8]: This section is not clear.

For the purpose of the evaluation of the A&T intervention on exclusive breastfeeding, sample size calculation required 30 mother-infant pairs where the infant is under 6 months per cluster (5), and the same sample size per cluster was used in other child age groups (6-11 and 12-23 months).

Data analysis

All analyses were conducted using STATA/SE version 14.1. Descriptive analyses were performed using sampling weights to account for sampling stratification by child's age group and accounted for the cluster sampling approach using the svy family of commands in STATA. Although none of the indicators related to IYCF practices and reported here were targeted by the A&T initiative, balance between trial arms was checked and findings are reported overall unless imbalance between trial arms was observed.

Commented [WU9]: ?

Indicators for IYCF practices, based on the 24-hour dietary recall and defined as per WHO's guidelines (7), included: i) ***Continued breastfeeding***: proportion of children who were reported to have been fed breastmilk; ii) ***Introduction of soft, semi-solid or solid foods***: proportion of children who were reported to have received at

least one soft, semi-solid or solid food; iii) ***Minimum dietary diversity***: proportion of children who were reported to have received foods from four or more different food groups out of seven food groups (defined as grains, roots and tubers; legumes and nuts; dairy; meat and fish; eggs; vitamin A-rich fruits and vegetables; other fruits and vegetables); iv) ***Minimum meal frequency***: proportion of children who were reported to have received soft, semi-solid, or solid foods (and milk feeds for non-breastfed children) at least twice for breastfed children aged 6 to 8 months, three times for breastfed children aged 9 to 23 months, and four times for non-breastfed children aged 6 to 23 months; v) ***Minimum milk feeding frequency***: proportion of non-breastfed children who were reported to have received at least two milk feeds; vi) ***Minimum acceptable diet***: proportion of children who were reported to have received the minimum acceptable diet, defined as at least both the minimum dietary diversity and the minimum meal frequency for breastfed children aged 6 to 23 months, or as at least 2 milk feeds, the minimum dietary diversity (dairy excluding) and the minimum meal frequency for non-breastfed children aged 6 to 23 months.

Other indicators related to IYCF practices included: i) Child feeding practices among children whose mother reported an episode of illness in the two weeks prior to interview; ii) Zinc supplementation among children whose mother reported an episode of diarrhoea in the two weeks prior to interview; iii) Vitamin A supplementation in the six months preceding the interview; iv) Proportion of children with their height, weight and mid-upper arm circumference (MUAC) measured during their last W-BC.

All indicators were computed by age group (children aged 6 to 11 months and 12 to 23 months). As per WHO's guidelines, continued breastfeeding was also calculated at 1 year (children aged 12 to 15 months) and at 2 years (children aged 20 to 23 months), and introduction of soft, semi-solid or solid foods was also calculated in children 6 to 8 months old (7).

Univariable and multivariable logistic regression was used to identify factors predictive of IYCF indicators. All regression models incorporated sampling weights to account for sampling stratification by child's age group, and commune and village as random effects to account for clustering. Household wealth index was computed from the first component of a Principal Component Analysis of 27 items collected from the household head. A score

was generated for correct knowledge of timely introduction of seven key food groups and was included as a linear term after checking for evidence of departures from linearity. Variables were initially selected for inclusion in the multivariable model if associated with the respective outcome variable with $p < 0.10$.

Ethical approval

Ethical approval was granted by the National Health Ethic Committee of the Ministry of Health of Burkina Faso (Reference 2015-5-061), the institutional review board of Centre MURAZ (Reference 2015-017) and the London School of Hygiene and Tropical Medicine (Reference 9066). Written informed consent was obtained prior to interview. Findings related to ethnicities are not shown to comply with the ethical requirement in Burkina Faso. The trial is registered at ClinicalTrials.gov (Reference NCT02435524).

Findings

Less than 1% of eligible mothers selected for interview were either absent, unable to participate or refused. A total of 2,229 women aged 15 to 49 years were interviewed, 1,116 had an infant aged 6 to 11 months and 1,113 had a child aged 12 to 23 months.

Women were 28 years old on average, from various ethnicities (data not shown), and predominantly of Muslim religion (63%) (table 1). Nearly all were in union (98%), predominantly in a monogamous union (65%). Around 30% of women and their husbands/partners had ever attended school. On average, women had had 3.9 live births and their youngest children were 14 months old.

IYCF practices

Based on mothers' reports, nearly all children (94%) were breastfed during the day or night prior to interview (table 2). Continued breastfeeding was almost universal (99%) among children aged 12 to 15 months and remained very high (77%) among children aged 20 to 23 months. Among children reported not to be breastfed, only 9% received the minimum milk feeding frequency (table 2).

About 70% of infants aged 6 to 11 months consumed soft, semi-solid or solid food (only 54% of infants aged 6 to 8 months), less than half (44%) received the minimum meal frequency, only 7% received the minimum dietary diversity, and only 6% received the minimum acceptable diet. Higher proportions of children 12 to 23 months met the criteria for minimum meal frequency (68%), minimum dietary diversity (24%) and minimum acceptable diet (17%).

Figure 1 shows child feeding patterns on the day and night prior to interview by age. The proportion of children who were breastfed and consumed soft, semi-solid or solid food increased from 29% at the age of 6 months to 82% or more from the age of 9 months. From 6 to 14 months old, the proportion of children who consumed breastmilk alone or with either plain water, milk or non-milk liquids decreased from 71% to 7%.

By far, the most commonly consumed food group was grains, roots and tubers (85%) (figure 2). Around half of children (53%) consumed vitamin A rich vegetables and fruits and about a third (37%) consumed iron rich foods (meat and fish). Each of the other food groups were consumed by about 15% or fewer children. The dietary diversity score, or mean number of food groups consumed, was 2.22 (95%CI 2.09, 2.36) in children aged 6 to 23 months: 1.33 (95%CI 1.20, 1.46) in infants aged 6 to 11 months compared to 2.65 (95%CI 2.49, 2.80) in children aged 12 to 23 months.

With respect to child feeding practices during an episode of illness, for only 9% and 20% of children aged 6 to 11 months and 12 to 23 months respectively did mothers report having increased breastfeeding or liquids and continued feeding (table 2).

Child growth monitoring, vitamin A and zinc supplementation

About three quarters of mothers (71%) reported having attended W-BC at least once since birth (table 1), but only 22% attended the last W-BC within the recommended time (within a month in children 6-11 months old and within two months in children 12-23 months old). At the last W-BC, height, weight and MUAC were reported to have been measured for 63%, 69% and 42% of children respectively (table 3).

Despite 59% of mothers reporting having brought their child to a health facility when suffering from diarrhoea in the two weeks prior to interview (table 1), only a third of children (31%) received zinc supplementation (table 3). With respect to vitamin A supplementation, 67% of mothers reported that their child had received a dose in the six months prior to interview.

Knowledge, attitudes and perceived self-efficacy

With respect to knowledge, some imbalances between trial arms were observed. Higher proportions of mothers in the intervention arm correctly stated that water or other liquids (88%) and porridge (80%) should be introduced at 6 months of age compared to 53% and 64% of mothers respectively in the control arm (table 4). Regarding other soft, semi-solid or solid foods, no substantial imbalances between trial arms were observed and 80% or more of mothers correctly stated that a child should be 6 months old or older (around 9 months on average) before dark green leafy vegetables, sweet potato, eggs or meat are introduced. However, among mothers who correctly stated that soft, semi-solid or solid foods should be introduced from the age of 6 months, 74% in the intervention arm had correct knowledge of minimum meal frequency compared to 64% in the control arm.

Knowledge of child feeding practices during illness was similar in both trial arms. While 85% of mothers knew that they should continue feeding their child when sick, fewer than a third correctly stated that a child should be breastfed (24%) or given liquids (30%) much more or more than usual (table 5).

With respect to attitudes, 70% or more of mothers were in agreement with statements that a child aged 6 to 8 months old who eats egg, meat, carrots/squash/sweet potato or dark green leafy vegetables will be healthy (table 6). However, among those who reported having introduced soft, semi-solid or solid foods, fewer felt capable of giving every day to their child dark green leafy vegetables (63%), meat, fish or egg (15%), or yellow/orange vegetables (12%).

Information on child complementary feeding

Despite relatively high proportions of mothers reporting contact with a health worker along the continuum of care (table 1), only 42% reported receiving information on child complementary feeding at a health facility in the control arm (table 7, figure 1 in supplementary material). More mothers, 66%, in the intervention arm reported receiving such information.

An imbalance between trial arms was also observed with respect to information received within the community with 25% and 41% of mothers in the control and intervention arms, respectively, reporting receiving such information (table 7, figure 1 in supplementary material). Relatives most commonly reported to have given information were the woman's mother (66%), followed by her mother-in-law (48%) and a sister (17%).

Predictors of IYCF practices

Child's age was a strong predictor of all IYCF practices with older children more likely to be appropriately fed: For instance, 10% of children 9-11 months benefited from the minimum dietary diversity compared to only 3% of children 6-8 months (OR = 4.63, 95%CI 2.48, 8.66) (table 8, tables 1 to 3 in supplementary materials). Other predictors of IYCF practices included: mother's ethnicity, mother's or partner's income generating activities, household wealth quintile, attendance to W-BC, having received information on child complementary feeding in the community and household time to water source. There was also strong evidence that having received information on child complementary feeding at a health facility and knowledge of the correct daily number of meals were associated with receiving the recommended minimum meal frequency (table 3 in supplementary materials).

Discussion

According to mothers' self-reports, only 18% and 13% of children aged 6 to 23 months living in rural areas of the Boucle du Mouhoun region benefited from the minimum dietary diversity and the minimum acceptable diet respectively. These findings reflect very poor IYCF practices and are similar to those reported from the 2016 Standardized Monitoring and Assessment of Relief and Transitions (SMART) survey in the same region (6). It should be noted, however, that the minimum dietary diversity reported at baseline in infants aged 6 to 11

months was slightly lower, at 2% (5), compared to 7% in this age group at endline. Both baseline and endline surveys were conducted between June and July and possible explanations for this include a secular trend towards improvement, a better harvest or a more detailed dietary recall at endline.

We also found poor levels of knowledge regarding timely introduction of complementary foods. Although most mothers reported that complementary foods should be introduced from 6 months of age or older, the average reported age for introduction was 9 months. Knowledge that a sick child should be breastfed or given liquids more than usual was particularly poor too. Interestingly, mother's perceived self-efficacy to provide children with key food groups on a daily basis was also relatively low compared to generally positive attitudes towards the introduction of these food groups.

Although the A&T initiative in the Boucle du Mouhoun region primarily targeted breastfeeding practices, some messages covered complementary feeding to respond to population demands on recommendations when children reach the age of 6 months. The imbalances observed between trial arms may reflect an effect of these messages. More women in the intervention arm reported having received information on IYCF practices when visiting a health facility or within the community. While knowledge of timely introduction of eggs, meat, dark green leafy vegetables and sweet potato were balanced between arms, women in the intervention arm reported better knowledge of timely introduction of water or other liquids, gruel/porridge and minimum meal frequency compared to women in the control arm. However, reported child complementary feeding in practices did not differ between arms.

Our study has some limitations. First, our findings are based on mothers' self-reports and, as in other similar studies, recall and social desirability bias cannot be excluded. Second, the survey took place at the beginning of the rainy season when food availability can be poor. IYCF practices may be better at other times of the year, for example after the harvest. Lastly, we cannot generalise our findings to other settings in Burkina Faso given substantial differences in IYCF practices between ethnicities.

Nevertheless, our findings highlight the need for interventions to improve mothers' knowledge and practices in relation to child complementary feeding in the Boucle du Mouhoun region. We found that women who reported attending W-BC were more likely to adopt recommended IYCF practices. Although exposure to facility-based information was only predictive of child's minimum meal frequency in our study, the lack of evidence for an association with other recommended practices should be interpreted bearing in mind that neither the quality nor the frequency of information received were collected and accounted for in our analyses. The high attendance of health facilities for W-BC, albeit not as regularly as recommended, represents an opportunity for contact with caretakers that should be exploited. Burkina Faso, however, suffers from understaffed and underresourced health centres and limited capacity of village health teams at the community level (8).

We also found evidence for an association of exposure to community-based information with IYCF practices, suggesting the potential of behaviour change communication strategies at the community level. Observed associations with mother's ethnicity suggests that social and cultural factors play an important role in nutrition. Qualitative data were collected during the trial and revealed that some "food taboos", such as not giving eggs to a child before s/he speaks, and a belief that solid food, if introduced too early, will delay the child's first steps may impede timely introduction of foods and dietary diversity (unpublished data). Interviews with mothers also revealed the influence of family members, in particular older women, and their role in supporting and relaying traditions has been reported in other settings, including Burkina Faso (9). Engagement and buy-in of other family members and other community members of influence is important to ensure effective change in social norms and behaviours.

A review of randomised and non-randomised controlled trials suggests that effective promotion of IYCF practices has the potential to increase good practices and child growth with stronger evidence for an effect in food insecure populations (10). In Bangladesh, Vietnam and Ethiopia, the A&T initiative combining facility- and/or community-based interpersonal communication, community mobilisation and mass media has been shown to be effective in improving reported IYCF practices. However, no intervention effect on child anthropometric outcomes was observed (11-14). In Hounde district in Burkina Faso, facility-based nutrition counselling from pregnancy until 18 months after birth increased reported recommended IYCF practices and child's birth weight but did not improve other child anthropometric measures (15).Possible explanations for the lack of effect on

child growth of these behaviour change communication strategies included only a small proportion of children benefiting from recommended IYCF practices at the end of the intervention, so that no effect on growth was detectable at the population level. Low health centre attendance and a relatively low coverage and intensity of the intervention was sometimes reported too. In some settings, food and resource availability may also have constrained sustained practices.

In our study, women living in poor households, who did not report an income generating activity or being in union with a partner earning an income in cash or kind were less likely to report the recommended practices. Burkina Faso ranked 185 out of 188 countries on UNDP's Human Development Index in 2014 (16), and 102 out of 118 countries on the Global Hunger Index (17) in 2016, with 16% of the population estimated to live with severe food insecurity (2). In this context, improving feeding practices and child growth may require more practical support such as social protection interventions.

Although evidence for the effect of nutrition-sensitive programs, such as integrated agriculture-nutrition or cash transfer interventions, is inconclusive or scarce (18), this type of intervention holds promise for improving both women and children's nutrition as well as women's empowerment. In Gourma province, eastern Burkina Faso, a two-year homestead food production program with bimonthly home visits improved child feeding practices with strong evidence for an effect on diarrhoea prevalence, weak evidence for an effect on wasting and haemoglobin level, but no evidence for an effect on stunting and underweight (19, 20). These mixed findings on child growth were discussed in light of the relatively short duration of the intervention and issues in the timing of its implementation. Some evidence for increased dietary diversity, lower prevalence of underweight and improved empowerment among mothers was also reported (21).

In Tapoa province, eastern Burkina Faso, unconditional cash transfers to mothers during the lean season increased children's intake of high-nutritional-value foods and the proportion receiving a minimum acceptable diet (22), but had no detectable effect on child growth (23). Qualitative and household expenditure data revealed that food and health were the first two investment domains for the cash received, not only for the

child, but for the whole family. The authors acknowledged the potential for a complementary behaviour change communication component to foster IYCF practices.

In conclusion, our findings revealed poor IYCF practices in rural areas of the Boucle du Mouhoun region with very few children benefiting from a minimum acceptable diet. Successful behaviour change communication strategies must be based on rigorous formative research in order to tailor the intervention to the local context. However, although these strategies have the potential to improve IYCF practices, comprehensive intervention packages that combine communication for behaviour change, increase coverage and quality of care and improvement in food security may be needed to foster and sustain change in feeding practices, and achieve optimal child growth.

Authors' contributions

VF, SC, SS, JAC, RG and AHD conceived the study design. SS, RG and HS designed the data collection instruments and implemented the survey with inputs from other authors. SS performed the analysis and wrote the initial draft of the manuscript. All authors reviewed, made inputs to and approved the final paper. VF is the overall guarantor and SS is the corresponding author.

Acknowledgments

We gratefully acknowledge all the fieldworkers, supervisors, and data managers for their work in the field. We also thank the study population for their participation.

Source of funding

Alive & Thrive is funded by the Bill & Melinda Gates Foundation and the governments of Canada and Ireland and managed by FHI 360. This research was supported by funding from the Bill & Melinda Gates Foundation, Grant Number OPP50838.

Conflict of interests

The authors have declared no competing interests.

396

Table 1: Socio-demographic characteristics of interviewed mothers and care seeking at a health facility along the continuum of care (N = 2,229)

		%	95%CI	
Mother's age	15-24 years	**37.5**	34.8	40.4
	25-34 years	**45.0**	42.7	47.3
	35-49 years	**17.5**	15.4	19.8
Mother's religion	Catholic/Protestant	**28.5**	22.1	35.9
	Muslim	**63.4**	54.7	71.3
	Animist/Atheist	**8.1**	4.7	13.4
Mother's education level	None	**73.2**	69.2	76.9
	Primary only	**18.0**	15.5	20.9
	Secondary or higher	**8.7**	7.0	10.7
Mother's income generating activities (cash or kind)		**57.9**	50.9	64.6
Mother's marital status	Monogamous union	**64.6**	61.1	68.0
	Polygamous union	**33.5**	30.0	37.0
	Single, divorced/separated, widow	**1.9**	1.3	2.8
Partner's education level	None	**66.5**	62.2	70.5
	Primary only	**23.7**	20.7	26.9
	Secondary or higher	**7.9**	6.3	9.9
	Not in union	**1.9**	1.3	2.8
In union with a partner earning an income in cash or kind		**81.7**	78.9	84.2
Parity (live births) (mean)		**3.9**	3.7	4.0
At least one antenatal (ANC) visit		**99.3**	98.2	99.7
4 or more ANC visits		**59.6**	55.0	64.0
Facility delivery		**89.9**	85.7	93.0
Postnatal care visit within one week of delivery (mother or baby)		**41.6**	34.2	49.4
Child's birth order	First live birth	**18.0**	16.3	19.9
	2nd or 3rd live birth	**31.9**	29.7	34.2
	4th to 6th live birth	**35.9**	34.0	37.8
	7th or above live birth	**14.2**	12.1	16.7
Child's gender	Boy	**52.5**	50.0	54.9
	Girl	**47.5**	45.1	50.0
Child's age	6-8 months	**17.7**	16.5	18.8
	9-11 months	**14.4**	13.3	15.5
	12-15 months	**27.2**	24.5	30.0
	16-19 months	**23.1**	20.9	25.5
	20-23 months	**17.6**	15.5	20.0
At least one well-baby consultation (W-BC) attendance since birth		**70.8**	63.8	76.9
Timely W-BC†		**21.8**	17.6	26.6

At least one visit to a health facility for immunisation since birth		**96.7**	95.1	97.8
Fever, cough, fast/difficult breathing or diarrhoea (past 2 weeks)		**37.0**	32.4	42.0
Consultation for childhood illness (sick children, past 2 weeks)		**60.9**	54.9	66.5
Consultation for diarrhoea (sick children, past 2 weeks)		**58.9**	51.3	66.2
Household clean water source*		**47.6**	38.8	56.6
Time from water source	In the yard	**10.7**	7.8	14.6
	< 10 minutes	**28.5**	25.0	32.3
	10 to 30 minutes	**41.7**	38.2	45.2
	> 30 minutes	**19.1**	16.0	22.7

* Public fountain, borehole, tap water; † Last WBC within 1 month if 6-11 months old or 2 months if 12-23 months old

Table 2: IYCF practices

On the day and night prior to interview	6-11 months (N = 715)			12-23 months (N = 1,514)			6-23 months (N = 2,229)		
	%	95%CI		%	95%CI		%	95%CI	
Continued breastfeeding	**99.8**	99.3	99.9	**91.9**	89.3	93.9	**94.4**	92.7	95.8
Introduction of soft/semi-solid/solid foods	**68.7**	64.3	72.8	**96.4**	95.0	97.4	**87.5**	85.5	89.3
Minimum meal frequency*	**44.4**	39.1	49.7	**67.5**	62.3	72.3	**59.8**	54.9	64.5
Minimum dietary diversity	**6.6**	5.1	8.5	**23.6**	19.7	28.1	**18.2**	15.2	21.6
Minimum acceptable diet*	**6.1**	4.6	8.0	**16.6**	13.1	20.9	**13.1**	10.4	16.4

	6-11 months (N = 1)			12-23 months (N = 122)			6-23 months (N = 124)		
	%	95%CI		%	95%CI		%	95%CI	
Minimum milk feeding frequency (non-breastfed children)	**0.0**	-	-	**8.9**	5.1	15.1	**8.8**	5.0	15.0

* excluding 258 mothers who did not know the number of times they had given soft, semi-solid, or solid foods to their child on the day prior to interview

During an episode of illness (2 weeks prior to interview)	6-11 months (N = 262)			12-23 months (N = 562)			6-23 months (N = 824)		
	%	95%CI		%	95%CI		%	95%CI	
Increased breastfeeding†	**10.5**	7.4	14.7	**12.6**	9.6	16.4	**11.9**	9.4	15.1
Increased liquids†	**17.1**	13.5	21.5	**25.2**	20.8	30.1	**22.6**	19.2	26.4
Increased breastfeeding or liquids†	**22.0**	17.8	26.9	**30.3**	25.7	35.2	**27.6**	24.1	31.5
Continued feeding††	**45.7**	39.0	52.6	**65.4**	59.1	71.2	**59.1**	54.0	64.1
Increased breastfeeding or liquids† & continued feeding††	**9.3**	6.3	13.4	**19.6**	15.5	24.5	**16.3**	13.1	20.2

† Much more or more than usual; †† Much more, more, as usual or slightly less than usual

Table 3: Child growth monitoring, vitamin A and zinc supplementation

	6-11 months (N = 715)	12-23 months (N = 1,514)	6-23 months (N = 2,229)

	%	95%CI		%	95%CI		%	95%CI	
Height measurement (last WBC)	**60.6**	52.3	68.3	**64.4**	57.2	71.1	**63.2**	55.8	70.0
Weight measurement (last WBC)	**66.7**	58.4	74.0	**70.0**	63.2	76.0	**68.9**	61.9	75.2
MUAC measurement (last WBC)	**40.8**	32.3	49.8	**42.7**	34.9	50.8	**42.1**	34.2	50.4
Height, weight & MUAC measurements (last WBC)	**39.3**	30.9	48.5	**41.2**	33.6	49.3	**40.6**	32.9	48.9
Vitamin A supplementation (past 6 months)	**65.1**	59.5	70.2	**68.6**	63.0	73.6	**67.4**	62.2	72.2

	6-11 months (N = 1[illegible]6)			12-23 months (N = 359)			6-23 months (N = 5[illegible]5)		
	%	95%CI		%	95%CI		%	95%CI	
Zinc supplementation (children with diarrhoea, past 2 weeks)	**27.6**	21.8	34.3	**35.3**	24.0	48.5	**30.8**	24.8	37.6

Table 4: Knowledge of timely introduction of complementary foods and minimum meal frequency

		Control arm (N = 1,150)			Intervention arm (N = 1,079)			Overall (N = 2,229)		
		%	95%CI		%	95%CI		%	95%CI	
**At what age should a mother start be giving her child:**										
Water or other liquids?	< 6 months	**42.0**	34.4	50.1	**7.6**	4.6	12.4	**25.4**	18.8	33.4
	6 months	**53.0**	45.1	60.8	**87.5**	82.3	91.3	**69.7**	61.8	76.6
	7 or 8 months	**2.0**	1.2	3.2	**3.6**	2.4	5.2	**2.7**	2.0	3.8
	> 8 months	**0.6**	0.2	1.5	**0.7**	0.4	1.5	**0.7**	0.4	1.2
	Does not know	**2.4**	1.3	4.5	**0.5**	0.3	1.2	**1.5**	0.8	2.7
	mean	**4.1**	3.6	4.5	**5.8**	5.6	5.9	**4.9**	4.5	5.3
Gruel/porridge?	< 6 months	**15.4**	11.6	20.2	**5.4**	3.4	8.7	**10.6**	7.9	14.0
	6 months	**64.3**	59.1	69.2	**80.2**	74.5	84.9	**72.0**	67.2	76.3
	7 or 8 months	**13.8**	11.8	16.2	**9.8**	7.5	12.9	**11.9**	10.2	13.9
	> 8 months	**4.7**	2.8	7.8	**3.4**	2.2	5.1	**4.1**	2.8	5.8
	Does not know	**1.7**	1.2	2.6	**1.2**	0.6	2.1	**1.5**	1.0	2.1
	mean	**6.1**	5.9	6.2	**6.1**	6.0	6.2	**6.1**	6.0	6.2
Dark green leafy vegetables*?	< 6 months	**5.2**	3.7	7.2	**1.3**	0.6	2.6	**3.3**	2.3	4.7
	6 months	**21.8**	18.5	25.5	**20.4**	16.3	25.1	**21.1**	18.5	24.1
	7 or 8 months	**26.4**	22.2	31.1	**28.1**	22.9	34.0	**27.2**	23.8	30.9
	> 8 months	**43.2**	38.2	48.4	**48.2**	43.9	52.5	**45.6**	42.2	49.1
	Does not know	**3.4**	2.1	5.3	**2.0**	1.2	3.4	**2.7**	1.9	3.9
	mean	**8.7**	8.4	9.0	**9.1**	8.8	9.3	**8.9**	8.7	9.1
	< 6 months	**4.6**	3.1	6.7	**1.3**	0.8	2.1	**3.0**	2.0	4.3

		%	95%CI		%	95%CI		%	95%CI	
Sweet potato?	6 months	**18.2**	15.5	21.2	**19.4**	15.4	24.1	**18.8**	16.3	21.5
	7 or 8 months	**23.5**	19.0	28.6	**27.6**	23.2	32.5	**25.5**	22.2	29.0
	> 8 months	**48.0**	42.4	53.6	**44.9**	41.6	48.3	**46.5**	43.2	49.9
	Does not know	**5.8**	4.3	7.8	**6.8**	4.7	9.6	**6.3**	5.0	7.9
	mean	**9.1**	8.9	9.4	**9.0**	8.8	9.2	**9.1**	8.9	9.2
Eggs?	< 6 months	**6.7**	4.6	9.8	**3.6**	2.4	5.3	**5.2**	3.9	7.0
	6 months	**24.9**	21.7	28.5	**31.3**	26.9	36.0	**28.0**	25.1	31.1
	7 or 8 months	**23.8**	20.8	27.0	**27.0**	22.8	31.7	**25.3**	22.7	28.2
	> 8 months	**32.9**	28.7	37.4	**26.4**	23.2	29.9	**29.8**	26.9	32.8
	Does not know	**11.6**	9.0	14.8	**11.7**	9.1	15.1	**11.7**	9.8	13.9
	mean	**8.3**	8.0	8.6	**8.0**	7.8	8.3	**8.2**	8.0	8.4
Meat?	< 6 months	**3.3**	2.2	4.9	**1.4**	0.8	2.2	**2.4**	1.7	3.3
	6 months	**13.3**	10.9	16.0	**13.8**	10.8	17.4	**13.5**	11.6	15.7
	7 or 8 months	**17.4**	14.3	21.0	**20.4**	18.1	22.9	**18.9**	16.8	21.1
	> 8 months	**59.3**	54.0	64.5	**59.1**	54.4	63.7	**59.2**	55.6	62.7
	Does not know	**6.7**	4.8	9.2	**5.3**	3.9	7.2	**6.0**	4.8	7.6
	mean	**9.9**	9.6	10.3	**9.8**	9.6	10.1	**9.9**	9.7	10.1
Soft, semi-solid, solid foods?	< 6 months	**5.9**	4.1	8.4	**1.4**	0.8	2.6	**3.7**	2.6	5.4
	6 months	**19.6**	16.0	23.7	**17.7**	12.7	24.0	**18.6**	15.5	22.3
	7 or 8 months	**24.9**	20.8	29.6	**28.4**	23.5	33.7	**26.6**	23.3	30.1
	> 8 months	**47.2**	41.8	52.6	**49.1**	42.9	55.3	**48.1**	44.0	52.2
	Does not know	**2.4**	1.5	3.9	**3.5**	2.5	4.8	**2.9**	2.2	3.9
	mean	**8.8**	8.4	9.1	**9.0**	8.7	9.4	**8.9**	8.7	9.1

* Baobab, sweet potato, cassava, black-eyed pea, moringa, spinach

	Control arm (N = 1,150)			Intervention arm (N = 1,079)			Overall (N = 2,229)		
	%	95%CI		%	95%CI		%	95%CI	
**From that age†, how many times a day should a mother feed her child with soft/semi-solid/solid foods?**									
2 times if 6-8 months, 3 times if 9-23 months	**63.9**	58.0	69.4	**73.8**	66.3	80.1	**68.8**	63.8	73.4
Does not know	**1.8**	1.0	3.3	**1.2**	0.7	2.0	**1.5**	1.0	2.3

† Referred to the age (6 months or above) given for the introduction of soft/semi-solid/solid foods

Table 5: Knowledge of child feeding practices during an episode of illness (N = 2,229)

	%	95%CI

**When a child is sick, should a mother breastfeed her child more, as usual or less?**	"much more" or "more"	**24.4**	21.7	27.3
	Does not know	**0.4**	0.2	0.8
**When a child is sick, should a mother give her child more liquids, as usual or less?**	"much more" or "more"	**30.9**	27.8	34.3
	Does not know	**0.3**	0.1	0.7
**When a child is sick, should a mother feed her child more, as usual or less?**	"much more, "more", "as usual" or "slightly less"	**85.0**	82.1	87.5
	Does not know	**0.5**	0.3	0.9

Table 6: Attitudes and perceived self-efficacy towards child complementary feeding

**Do you agree with the following statement? (N = 2,229)**		%	95%CI	
"A child aged 6 to 8 months old who eats egg will be healthy"	Agree	**83.4**	79.9	86.4
	Does not know	**4.4**	3.3	5.9
"A child aged 6 to 8 months old who eats meat will be healthy"	Agree	**71.8**	66.6	76.6
	Does not know	**4.0**	2.9	5.5
"A child aged 6 to 8 months old who eats carrot/squash/sweet potato will be healthy"	Agree	**76.1**	71.3	80.3
	Does not know	**5.0**	3.8	6.6
"A child aged 6 to 8 months old who eats dark green leafy vegetables* will be healthy"	Agree	**81.1**	77.6	84.2
	Does not know	**2.8**	1.9	3.9

**Do you feel capable of giving every day to your child (mothers who introduced soft, semi-solid, solid foods, N = 1,889)?**	%	95%CI	
Meat, fish or egg	**14.6**	12.2	17.4
Dark green leafy vegetables*	**62.9**	57.9	67.6
carrot/squash/sweet potato	**11.2**	9.0	14.0

*Baobab, sweet potato, cassava, black-eyed pea, moringa, spinach

Table 7: Information on child complementary feeding received at a health facility and in the community

	Control arm (N = 1,150)			Intervention arm (N = 1,079)			Overall (N = 2,229)		
	%	95%CI		%	95%CI		%	95%CI	
At any point of care*	**41.9**	34.3	49.8	**65.6**	57.4	73.0	**53.4**	46.5	60.1
At any place in the community**	**24.5**	19.2	30.7	**40.6**	34.9	46.6	**32.3**	27.5	37.4
At any point of care* or place in the community**	**52.2**	44.7	59.6	**73.8**	68.2	78.7	**62.7**	56.6	68.4

* During a consultation for childhood illness, a visit for child growth monitoring or for immunisation, or during a group discussion

** During a group discussion or a home visit, by a local healer or a relative, by listening to radio, or at other occasions

Table 8: Predictors of minimum acceptable diet (MAD) in children 6 to 23 months of age (N = 1,971)

		N	MAD %	Univariable				Multivariable			
				OR	95%CI		P-value	OR	95%CI		P-value
Mother's age	15-24 years	760	12.7	**1.00**	-	-	0.536				
	25-34 years	876	12.8	**1.02**	0.75	1.39					
	35-49 years	334	14.9	**1.24**	0.83	1.85					
Mother's ethnicity*							0.005				< 0.001
Mother's religion	Catholic/Protestant	574	10.9	**1.00**	-	-	0.151				
	Muslim	1,254	14.4	**1.26**	0.73	2.15					
	Animist/Atheist	143	10.3	**0.72**	0.37	1.39					
Mother's education level	None	1,422	13.5	**1.00**	-	-	0.527				
	Primary only	364	13.0	**0.97**	0.63	1.48					
	Secondary or higher	185	10.6	**0.71**	0.40	1.28					
Mother's income generating activities (cash or kind)	No	812	9.5	**1.00**	-	-	0.007	**1.00**	-	-	0.101
	Yes	1,159	15.6	**1.65**	1.15	2.37		**1.41**	0.94	2.13	
Mother's marital status	Monogamous union	1,302	13.4	**1.00**	-	-	0.576				
	Polygamous union	630	13.1	**0.96**	0.67	1.37					
	Single, separated, widow	39	6.9	**0.45**	0.10	2.02					
Partner's education level	None	1,299	13.4	**1.00**	-	-	0.720				
	Primary only	474	13.0	**1.02**	0.66	1.56					
	Secondary or higher	159	12.7	**1.08**	0.55	2.12					
	Not in union	39	6.9	**0.46**	0.10	2.04					
In union with a partner earning an income in cash or kind	No	370	6.0	**1.00**	-	-	0.001	**1.00**	-	-	0.012
	Yes	1,601	14.8	**2.69**	1.54	4.69		**2.30**	1.20	4.44	
4 or more ANC visits	No	795	13.0	**1.00**	-	-	0.896				
	Yes	1,176	13.2	**0.98**	0.67	1.41					
Facility delivery	No	195	10.4	**1.00**	-	-	0.928				
	Yes	1,776	13.4	**1.04**	0.43	2.54					
Postnatal care visit within 1 week of delivery (mother or baby)	No	1,175	12.5	**1.00**	-	-	0.470				
	Yes	796	14.1	**1.12**	0.82	1.53					
Child's birth order	First live birth	362	14.2	**1.00**	-	-	0.884				
	2nd or 3rd live birth	640	11.8	**0.88**	0.57	1.35					
	4th to 6th live birth	703	14.0	**1.03**	0.75	1.42					
	7th or above live birth	265	12.5	**0.96**	0.55	1.68					

Child's gender	Boy	1,045	12.5	**1.00**	-	-	0.354				
	Girl	926	13.9	**1.15**	0.86	1.54					
Child's age	6-8 months	369	2.8	**1.00**	-	-	< 0.001	**1.00**	-	-	< 0.001
	9-11 months	284	10.4	**4.81**	2.63	8.78		**4.63**	2.48	8.66	
	12-15 months	531	13.3	**6.08**	3.69	10.03		**5.80**	3.54	9.49	
	16-19 months	446	19.2	**9.70**	5.66	16.62		**10.27**	5.80	18.17	
	20-23 months	342	18.3	**9.67**	5.20	17.98		**8.94**	4.90	16.32	
Fever, cough, fast/difficult breathing or diarrhoea (past 2 weeks)	No	1,253	12.8	**1.00**	-	-	0.311				
	Yes	717	13.7	**1.19**	0.85	1.65					
At least one well-baby consultation (W-BC) attendance since birth	No	579	8.7	**1.00**	-	-	0.001	**1.00**	-	-	0.012
	Yes	1,392	15.0	**2.09**	1.36	3.21		**1.93**	1.16	3.23	
At least one visit to a health facility for immunisation since birth	No	68	2.9	**1.00**	-	-	0.159				
	Yes	1,903	13.5	**3.13**	0.64	15.34					
Received facility-based information on complementary feeding	No	910	10.6	**1.00**	-	-	0.202				
	Yes	1,060	15.3	**1.30**	0.87	1.95					
Received communitybased information on complementary feeding	No	1,324	10.7	**1.00**	-	-	0.001	**1.00**	-	-	0.023
	Yes	647	18.1	**1.79**	1.26	2.53		**1.57**	1.06	2.31	
Household wealth quintile	Poorest	401	8.5	**1.00**	-	-	0.033	**1.00**	-	-	0.054
	Poorer	387	11.4	**1.35**	0.72	2.52		**1.28**	0.65	2.52	
	Middle	375	11.2	**1.13**	0.61	2.06		**0.94**	0.51	1.75	
	Richer	393	16.3	**1.92**	1.11	3.30		**1.67**	0.91	3.07	
	Richest	409	18.0	**2.15**	1.15	4.02		**1.97**	1.00	3.86	
Household clean water source**	No	1,020	10.8	**1.00**	-	-	0.032	**1.00**	-	-	0.348
	Yes	951	15.7	**1.46**	1.03	2.06		**1.17**	0.84	1.63	
Time from water source	> 30 minutes	389	11.8	**1.00**	-	-	0.387				
	10 to 30 minutes	828	12.1	**1.13**	0.72	1.77					
	< 10 minutes	754	14.9	**1.43**	0.86	2.38					

* Data not shown to comply with the ethical requirement in Burkina Faso

** Public fountain, borehole, tap water

References

1. Black RE, Victora CG, Walker SP, Bhutta ZA, Christian P, de Onis M, Ezzati M, et al. Maternal and Child Undernutrition and Overweight in Low-Income and Middle-Income Countries. *Lancet* 2013; 382: 427-51.
2. Food and Agriculture Organisation of the United Nations, International Fund for Agricultural Development, UNICEF, World Food Programme and World Health Organisation. The State of Food Security and Nutrition in the World. Building resilience for peace and food security. FAO (Rome); 2017.
3. You D, Hug L, Ejdemyr S, Idele P, Hogan D, Mathers C, Gerland P, New JR, Alkema L, United Nations Interagency Group for Child Mortality Estimation (UN IGME). Global, regional, and national levels and trends in under-5 mortality between 1990 and 2015, with scenario-based projections to 2030: a systematic analysis by the UN Inter-agency Group for Child Mortality Estimation. *Lancet* 2015; 386: 2275-86.
4. Cresswell JA, Ganaba R, Sarrassat S, Some H, Diallo AH, Cousens S, Filippi V. Effect of the Alive & Thrive initiative to increase exclusive breastfeeding in Burkina Faso: a cluster randomised controlled trial. *The Lancet Global Health* 2019; : e357-e365.
5. Cresswell JA, Ganaba R, Sarrassat S, Cousens S, Some H, Diallo AH, Filippi V. Predictors of exclusive breastfeeding and consumption of soft, semi-solid or solid food among infants in Boucle du Mouhoun, Burkina Faso: A cross-sectional survey. *PLoS One* 2017; 12: e0179593.
6. Ministère de la Santé, Burkina Faso. Enquête nutritionnelle nationale 2016. Ministère de la santé (Ouagadougou); 2016. Available from: https://www.humanitarianresponse.info/system/files/documents/files/smart_2016.pdf (Accessed Dec 22, 2017)
7. World Health Organisation. Indicators for assessing infant and young child feeding practices. Part 1: definitions. Conclusions of a consensus meeting held 6-8 November 2007 in Washington, DC, USA. WHO (Geneva); 2008.
8. Murphy E, Oot L, Sethuraman K. USAID Office of Food for Peace Food Security Desk Review for Burkina Faso. Washington, DC: FHI 360/FANTA. 2017.
Available from: https://www.fantaproject.org/sites/default/files/resources/FFP-Burkina-Faso-Desk-Review-

Oct2017.pdf (Accessed May 24, 2018)

9. Aubel J. The role and influence of grandmothers on child nutrition: culturally designated advisors and caregivers. *Matern Child Nutr* 2012; 8: 19-35.

10. Lassi ZS, Das JK, Zahid G, Imdad A, Bhutta ZA. Impact of education and provision of complementary feeding on growth and morbidity in children less than 2 years of age in developing countries: a systematic review. *BMC Public Health* 2013; 13: S13.

11. Sanghvi T, Haque R, Roy S, Afsana K, Seidel R, Islam S, Jimerson A, Baker J. Achieving behaviour change at scale: Alive & Thrive's infant and young child feeding programme in Bangladesh. *Matern Child Nutr* 2016; 12: 141-54.

12. Menon P, Nguyen PH, Saha KK, Khaled A, Sanghvi T, Baker J, Afsana K, Hague R, Frongillo EA, Ruel MT, Rawat R. Combining intensive counselling by frontline workers with a nationwide mass media campaign has large differential impacts on complementary feeding practices but not on child growth: results of a clusterrandomised program evaluation in Bangladesh. *J Nutr* 2016; 146: 2075-84.

13. Kim SS, Rawat R, Mwangi EM, Tesfaye R, Abede Y, Baker J, Frongillo EA, Ruel MT, Menon P. Exposure to largescale social and behaviour change communication interventions is associated with improvements in infant and young child feeding practices in Ethiopia. *PLoS One* 2016; 11: e0164800.

14. Rawat R, Nguyen PH, Tran LM, Hajeebhoy N, Nguyen HV, Baker J, Frongillo EA, Ruel MT, Menon P. Social franchising and nationwide mass media campaign increased the prevalence of adequate complmentary feeding in Vietnam: a cluster-randomised program evaluation. *J Nutr* 2017; 147: 670-79.

15. Nikiema L, Huybregts L, Martin-Prevel Y, Donnen P, Lanou H, Grosemans J, Offoh P, Dramaix-Wilmet M, Sondo B, Roberfroid D, Kolsteren P. Effectiveness of facility-based personalised maternal nutrition counseling in improving child growth and morbidity up to 18 months: a cluster-randomised controlled trial in rural Burkina Faso. *PLoS One* 2017; 12: e0177839.

16. Jahan S. Human Development Report 2016: Human Development for Everyone. UNDP (New York); 2016. Available from: http://hdr.undp.org/en/2016-report (Accessed Dec 22, 2017)

17. von Grebmer K, Bernstein J, Prasai N, Amin S, Yohannes Y. 2016 Global Hunger Index: Getting to zero hunger.

Bonn (Washington); 2016. Co-published by the International Food Policy Research Institute and Concern Worldwide. Available from: http://www.ifpri.org/publication/2016-global-hunger-index-getting-zerohunger (Accessed Dec 15, 2017)

18. Ruel MT, Alderman H; Maternal and Child Nutrition Study Group. Nutrition-sensitive interventions and programmes: how can they help to accelarate progress in improving maternal and child nutrition. *Lancet* 2013; 382: 536-51.
19. Olney DK, Pedehombga A, Ruel MT, Dillon A. A 2-year integrated agriculture and nutrition and health behaviour change communication program targeted to women in Burkina Faso reduces anemia, wasting, and diarrhea in children 3-12.9 months of age at baseline: a cluster-randomised controlled trial. *J Nutr* 2015: 145: 1317-24.
20. Heckert J, Olney DK, Ruel MT. Is women's empowerment a pathway to improving child nutrition outcomes in nutrition-sensitive agriculture program?: Evidence from a randomised controlled trial in Burkina Faso. *Soc Sci & Med* 2019; 233: 93-102.
21. Olney DK, Bliznashka L, Pedehombga A, Dillon A, Ruel MT, Heckert J. A 2-year integrated agriculture and nutrition program targeted to mothers of young children in Burkina Faso reduces underweight among mothers and increases their empowerment: a cluster-randomized controlled trial. *J Nutr* 2016: 146: 1109-17.
22. Tonguet-Papuci A, Houngbe F, Huybregts L, Ait-Aissa M, Altare C, Kolsteren P, Huneau JF. Unconditional seasonal cash transfer increases intake of high-nutritional-value foods in young Burkinabes children: results of 24-hour dietary recall surveys within the MAM'Out randomised controlled trial. *J Nutr* 2017; 147: 1418-25.
23. Houngbe F, Tonguet-Papucci A, Altare C, Ait-Aissa M, Huneau JF, Huybregts L, Kolsteren P. Unconditional cash transfers do not prevent children's undernutrition in the Moderate Acute Malnutrition Out (MAM'Out) cluster-randomised controlled trial in rural Burkina Faso. *J Nutr* 2017; 147: 1410-17.

Figure

Click here to download Figure po_iycf practices_v5_fig.docx

Figure 1: Child feeding patterns by age during the day and night prior to interview (N = 2,229)

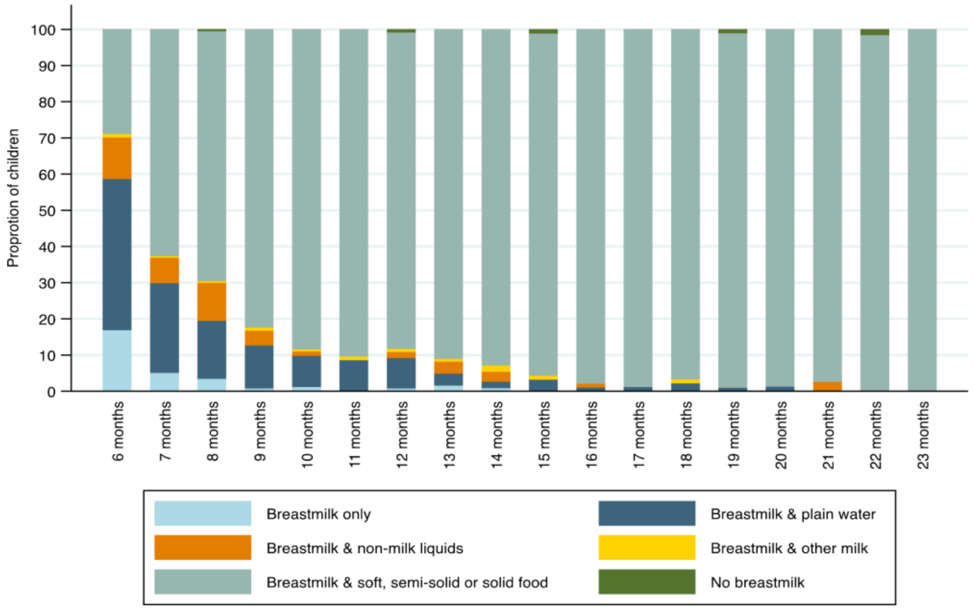

Categories are hierarchical & mutually exclusive
Based on 24-hour dietary recall

Figure 2: Children dietary diversity and consumption of the seven food groups during the day and night prior to interview (N = 2,229)

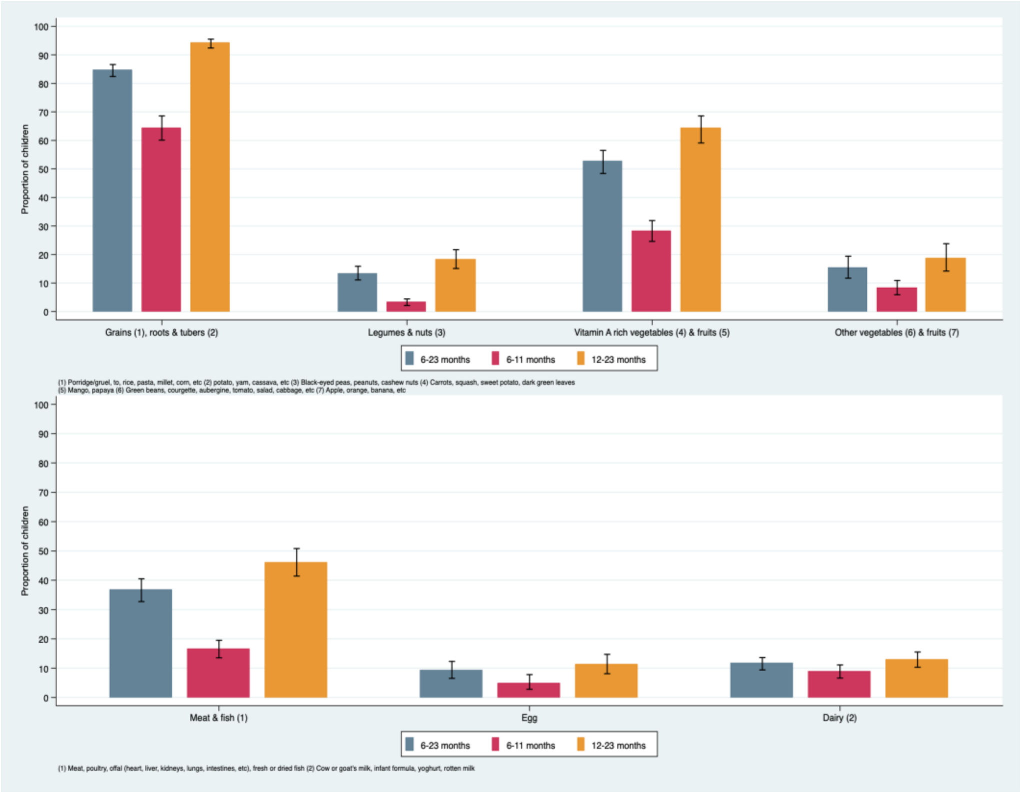

Supporting Information

Click here to access/download
Supporting Information
po_iycf
practices_v5_sup.docx
